# A rare finding on computed tomography angiography performed to exclude pulmonary embolism

**DOI:** 10.1007/s12471-022-01744-1

**Published:** 2022-12-12

**Authors:** Vivan J. M. Baggen, Léon J. P. M. van Woerkens, Robert M. Kauling, Atilla Dirkali

**Affiliations:** 1 Albert Schweitzer Ziekenhuis, Dordrecht, The Netherlands; 2 Erasmus Medical Centre, Rotterdam, The Netherlands

## Answer

**Fig. 1:**
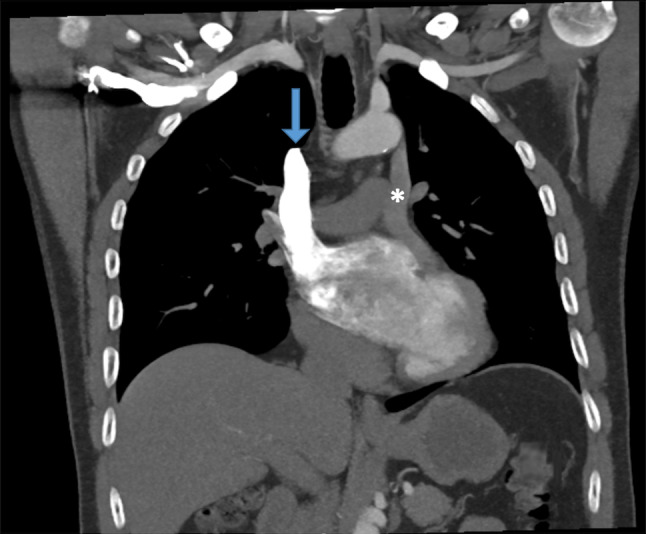
Computed tomography angiography, coronal plane. *Arrow* indicates right superior vena cava (*SVC*) with contrast, draining into the left atrium. *Asterisk* indicates persistent left SVC, draining into a dilated coronary sinus (which is also shown in Fig. 1a of the Question)

The right superior vena cava (SVC) drains into the left atrium (Fig. 1, arrow). This would normally cause hypoxaemia due to right-left shunting, as the venous return from the entire upper body is shunted to the systemic circulation. However, this patient also has a persistent left SVC, which drains via the coronary sinus into the right atrium (Fig. 1, asterisk). Hence, only the venous return of the right upper hemithorax is shunted. On reassessment, echocardiography was found to show a dilated coronary sinus, which is a common finding in persistent left SVC.

Isolated persistent left SVC occurs in ~ 0.3–0.5% of the general population. Persistent left SVC is associated with other cardiovascular abnormalities in 40% of cases [1]. Isolated right SVC drainage into the left atrium is extremely rare. Approximately 20 cases have been reported, mostly in children presenting with mild hypoxaemia and cyanosis. The current combination of right SVC drainage into the left atrium and persistent left SVC has been reported in only three patients [2, 3]. Its incidence is possibly underestimated due to the lack of clinical symptoms.

The patient was referred to a tertiary centre for congenital heart disease. On re-evaluation the patient reported no palpitations and no limitation of ordinary physical activity. Exercise testing including saturation sampling showed an arterial oxygen saturation at rest of 96%, with a gradual decrease during exercise to 93%. Because of the limited clinical symptoms, there was no indication for surgical correction. Future venous injections via the right arm should be avoided, to prevent systemic embolism.
